# Outdoor recreation’s association with mental health and well-being during the COVID-19 pandemic

**DOI:** 10.1371/journal.pone.0321278

**Published:** 2025-04-17

**Authors:** Colby Parkinson, Xiangyou Shen, Megan MacDonald, Samuel W. Logan, Lydia Gorrell, Kreg Lindberg

**Affiliations:** 1 Department of Forest Ecosystems and Society, Oregon State University, Corvallis, Oregon, United States of America; 2 Department of Recreation, Park, and Tourism Management, Pennsylvania State University, University Park, Pennsylvania, United States of America; 3 College of Health, Oregon State University, Corvallis, Oregon, United States of America; UCL: University College London, UNITED KINGDOM OF GREAT BRITAIN AND NORTHERN IRELAND

## Abstract

Outdoor recreation provided a crucial way to maintain physical activity, reduce stress, and preserve a sense of normalcy during the COVID-19 pandemic. This study assessed the relationship between outdoor recreation and mental health in the context of COVID-19. Cross-sectional online survey data were collected in early 2021 from a sample (*n* = 503) representative of the U.S. adult population in age, gender, and race. We observed prevalent engagement in near-home outdoor activities, widespread reductions in outdoor engagement relative to the pre-COVID period, and significant age, financial, and racial differences in engagement patterns. Regression models suggested that reduced outdoor recreation was associated with higher levels of perceived stress and depressive symptoms, whereas more frequent outdoor activities predicted better well-being. The health implications of adaptive engagement versus cumulative exposure during times of significant disruptions are discussed, along with the need to address structural inequities in accessing outdoor recreation as a health behavior.

## Introduction

The COVID-19 pandemic caused a mental health crisis [1], increasing the worldwide prevalence of anxiety and depression by 25% [2]. In the U.S., the beginning lockdown phase witnessed as high as three-fold increases in stress, anxiety, and depression among the general population [3,4], a trend that persisted after COVID-19 vaccine approvals [5–7]. As access to out-of-home indoor spaces instrumental to personal and community functioning drastically declined, people around the world increasingly turned to outdoor natural areas as relatively safe spaces for recreation and restoration [8–13]. While this shift coincided with greater gender, ethnic, age, and financial diversity among new outdoor recreation participants [14], historical inequities that exclude or constrain use of outdoor spaces were exacerbated during the pandemic [15], particularly among women, racial and ethnic minorities, and low income individuals in the U.S. [9,16,17].

Understanding nature exposure patterns during COVID-19 is important because, consistent with long-standing research that supports the physical and mental health benefits of nature exposure [18,19], nature contact (e.g., gardening, visiting green or blue spaces) helped mitigate the negative effects of pandemic stressors on numerous mental and physical health outcomes [20,21]. Specifically, more nature contact, whether indicated by cumulative exposure measures (e.g., frequency, duration) or relative changes compared to pre-COVID level, was associated with lower perceived stress [10,22–24], reduced depressive symptoms [25–27], and better overall mental health and well-being [24,28,29]. In this study, we aimed to contextualize outdoor recreation engagement during COVID-19 and develop a better understanding of its potential mechanism for promoting mental health in times of severe disruptions and constraints.

### Outdoor recreation during the height of the COVID-19 pandemic

Throughout different phases of the pandemic, significant variations existed in outbreak levels and pandemic responses across countries and locales, shaping diverse outdoor space policies, public health measures and guidance, and individual adherence to preventive measures and perceptions of risk [12,13,30,31]. At a high-level, many countries first implemented legally restrictive measures (e.g., stay-at-home, ban on large gatherings) and public health guidance to reduce the spread of the virus in March 2020, often removing, adjusting, and re-implementing measures depending on the severity of nationwide or local spread through the remainder of 2020 and beyond [32]. Throughout this period, many governments instituted complete or partial lockdowns that inhibited or discouraged the use of public and recreational spaces [12]. In 2021, the approval of vaccines alongside more infectious and deadly local outbreaks from different COVID-19 variants brought greater variability in pandemic response. Wealthy countries rolled out millions of vaccines that protected citizens from more deadly outcomes while broadly ending or reducing restrictive measures, although many poor countries or vulnerability communities continued to suffer [33].

Mixed evidence was reported regarding the rate of outdoor recreation and use of outdoor spaces during COVID-19. Compared to pre-pandemic levels, decreased outdoor recreation and facility use were observed in places that implemented park and recreation facility closure or other lockdown measures [23,36,37]. Meanwhile, many regions or recreational facilities that implemented minimal or eased restrictions experienced increased visitation [12,38,39], particularly at facilities with more natural features and those nearest to people’s homes [40,41]. Notably, several studies observed increases in neighborhood walking or jogging [42–44], reliance on at-home nature and gardening [45,46], and many other less specialized outdoor activities [34,47]. These activities are typically excluded from outdoor recreation taxonomies commonly used in outdoor participation surveys and studies examining recreation facility usages. Overall, our literature review revealed largely inconsistent findings about individual-level outdoor recreation participation during the pandemic [10,24,28,42,48,49]. Many factors might have contributed to these inconsistencies: diverse research designs, different outdoor space uses as a result of heterogeneous time- and place-related pandemic responses at both the individual and societal level [20,34,35,50], seasonal variations associated with regional climates [37], and structural inequities and individual differences associated with demographic characteristics [9,16,51].

In this study, we examine the outdoor recreation rate in the U.S. in early 2021, one of the country’s most deadly and infectious periods [52]. Relative to other countries, the U.S. took a localized approach to managing the pandemic [31] and, at the time of the study, implemented no nationally instituted laws that restricted mobility, the latter often associated with decreased outdoor recreation [53,54]. Some research observed higher-than-pre-pandemic annual participation rate (54%) in outdoor recreation in 2021 [55]. Other studies observed increased [56] or decreased [39] outdoor recreation during later phases of the pandemic. Through this study, we aimed to collect data on the broad patterns of outdoor engagement during a later phase of the pandemic to reveal emergent popular activities and potential demographic differences that are underexamined in existing research. Instead of using a pre-defined set of outdoor recreation activity categories, respondents in our study self-nominated their most frequent outdoor recreation activity. This approach ensures the inclusion of a broader set of activities pursued by people in an outdoor setting for recreational purposes.

### Outdoor recreation and mental health

Outdoor recreation provides a direct and engaging form of nature contact because it usually involves physical movement and close interactions with the natural environment [19]. However, different measures of outdoor recreation engagement and broader nature exposure have been associated with varied benefits to mental health outcomes during the COVID-19 pandemic [21]. Existing research suggested that increasing or maintaining time spent outside during the pandemic was associated with lower perceived stress and higher positive mental health [23,24,56], whereas decreasing time outside was associated with higher depression [48]. Additionally, people who maintained pre-COVID levels of outdoor recreation experienced smaller decreases in subjective well-being than those who reduced outdoor activities [28], potentially revealing an adaptive effect of outdoor recreation. Compared to indoor space use, outdoor space use during COVID-19 was more strongly associated with well-being [57], reinforcing outdoor space’s contribution to adaptation, especially as people intentionally used these spaces to improve their health outcomes [43,51].

Studies have observed that, regardless of the relative levels of outdoor recreation compared to the pre-COVID period, cumulative exposure as indicated by outdoor recreation frequency was associated with higher well-being [21,29,58] and reduced depression [25–27]. Furthermore, improved well-being was associated with spending any amount of time outdoors relative to non-participation [8]. These behavior-focused findings are consistent with the observations from outdoor space use studies, which found both proximity to green spaces and actual use contributed to improved mental health and engagement in healthy behaviors [59–61].

Despite thorough examination of their independent effects, few studies included both relative and cumulative measures when examining outdoor recreation’s association with mental health during COVID-19 or in other contexts. It remains unclear which measure is a better predictor of mental health in times of major disruption and to what extent one may confound the other. Previous research that examined leisure engagement and mental health during the pandemic revealed that these two types of measures were related but not equivalent, and, in times of significant interruptions, adaptive engagement indicated by relative measures may better predict mental health [62]. In this study, we include both types of measures to enable comparison and examine outdoor recreation’s potentially adaptive role during stressful events.

Most existing literature on outdoor recreation and mental health also focused on early stages of the pandemic, particularly the lockdown phase [24,26,28]. While later-phase studies generally reported consistent evidence in favor of nature’s contribution to health [59], their focus was on different regions or populations than the present study. In general, several studies examined European countries [8,57,59] or specific regions or destinations within the U.S. [29,60,63]. Others sampled only specific groups, such as college students [10,58,64]. In this study, we seek to expand our understanding on the outdoor recreation-mental health relationship beyond early stages of the pandemic using data collected from a diverse nationwide U.S. adult sample.

### COVID-specific risk and protective factors

Research has identified salient risk factors that might worsen mental health during COVID-19, including higher perceived risk of infection [3,65] and loneliness or social isolation [66, 67]. Negative perceptions and beliefs regarding preventive measures have also been associated with poorer mental health [62,68]. On the flip side, positive beliefs about preventive measures might have a protective effect on mental health. Engaging in precautionary behavior intended to reduce the risk of infection (e.g., wearing a mask, washing hands) has been identified as a protective mental health factor [69,70]. Additionally, more positive future outlooks that were measured using optimism [71], hope [70], and anticipation of a better world [72] were associated with better mental health outcomes during the pandemic.

Researchers have identified risk and protective factors in the context of nature exposure and mental health during COVID-19 at the environmental and community levels, such as severity of lockdowns [23,24] and COVID-19 death rate [10], and related to individuals’ lifestyles, such as screen time [64,73] and physical activity [24,48,58]. Findings from different countries and pandemic stages suggested that risk and protective factors might confound the relationship between nature contact and health outcomes [23,27,64].

In this study, we focus on individual-level risk and protective factors as informed by previous research, including infection risk perceptions, social isolation, beliefs about preventive measures, practices of precautionary behavior, and future outlook about vaccines and a post-pandemic world. These factors are included as covariates to control for their possible confounding effect on the association between outdoor recreation and mental health.

### Research gaps and questions

The current study extends previous literature in five key ways: first, it collected data from a nationwide sample to reveal broad patterns of outdoor recreation among U.S. adults with a focus on an individual’s most frequent outdoor activity, as the latter may provide an anchor and special meaning through a time of turmoil and disruptions. Second, we let participants self-define outdoor recreation to allow for the inclusion of key outdoor activities that became prevalent during the pandemic but would have been excluded from a conventional definition or activity taxonomy [47,74]. Third, this study was conducted during a period that overlapped with one of the largest waves of COVID-19 cases and deaths in the U.S. [52], providing a unique opportunity to examine the outdoor recreation-mental health relationship under severe and sustained stress. Fourth, unlike most existing studies that used either cumulative measures (e.g., frequency, duration) or relative measures of outdoor recreation (e.g., changes relative to pre-COVID level), we included both to enable comparisons and insights on the possibly unique effect of changes in outdoor engagement on mental health independent of the impact of cumulative exposure. Fifth, when analyzing the outdoor recreation-mental health relationship, we minimize biases by controlling for potential confounding COVID-specific risk and protective factors. The latter has been accounted for by only a small number of previous studies [21,48,58,64].

To address the above gaps, we asked two research questions:

(1) What were the broad patterns of outdoor recreation, including variety of most frequent activity, relative change, frequency, and demographic group differences, among U.S. adults nationally during the pandemic?
(2) What was the association between outdoor recreation engagement and mental health and well-being, controlling for COVID-specific risk and protective factors?

## Methods

Data in this study were collected from a nationwide sample proportionally representative of the U.S. adult population in age, gender, and race (*n* = 503). Participants were recruited via Prolific, an online crowd-sourcing platform, and screened to meet two inclusion criteria: aged 18 years or older and residing in the U.S. at the time of survey. A Qualtrics survey was administered between February 3 and February 15, 2021. The study was approved by the Institutional Review Board of the authors’ institution (IRB-2020–0927). The research was deemed to pose minimal risk with the only potentially identifiable information collected being zip code. All participants provided written consent that was recorded via the online survey.

This research was part of a larger study investigating leisure engagement patterns among U.S. adults. We aimed to collect data from a national sample representing diverse backgrounds. A previous cross-sectional study on health during COVID-19 [30] using the same platform demonstrated that a sample size of 500 was sufficient to ensure diverse socio-demographic representations. Power analyses (assuming medium effect size, 95% CI, and α =.05) confirmed this exceeded the minimum required sample size for each planned statistical test. More details about the research design and sampling method are reported in prior published research [62].

### Instrumentation

#### Mental health and well-being outcomes.

Perceived stress was measured using the 7-item stress subscale from the Depression, Anxiety, and Stress Scale (DASS-21) [75, 76]. Participants used a 4-point scale (0 = “Not at all” to 3 = “Most of the time”). Participants reported their agreement with how often statements regarding difficulty relaxing and general agitation (e.g., “I found it hard to wind down” and “I tended to over-react to situations”) applied to how they felt over the last two weeks. Responses were summed for final analysis and ranged from 0 to 21 with higher scores signifying higher perceived stress. The scale had excellent internal reliability (Cronbach’s α =.92).

Depressive symptoms were measured using the Patient Health Questionnaire (PHQ-2) [77]. Participants used a 4-point scale (0 = “Not at all” to 3 = “Nearly every day”) to report how often they were bothered by “Little interest or pleasure in doing things” and “Feeling down, depressed, or hopeless” over the last two weeks. Responses were summed and ranged from 0 to 6 with high scores signifying more depressive symptoms. The scale had good internal reliability (Cronbach’s α =.85).

Well-being was measured using the World Health Organization Well-being Index (WHO-5) [78]. Participants used a 5-point scale (0 = “At no time” to 4 = “All of the time”) to report how frequently statements regarding positive mood and vitality (e.g., “I have felt cheerful and in good spirits” and “I have felt calm and relaxed”) applied to how they felt over the past two weeks. Responses were summed and ranged from 0 to 25 with higher scores indicating better well-being. The scale had excellent internal reliability (Cronbach’s α =.92).

#### Outdoor recreation engagement.

Participants reported their most frequent outdoor recreation activity during COVID-19 through an open-ended question. Two additional questions using 5-point scales covered past-month outdoor recreation frequency (1 = “Less than once a week,” 2 = “Once a week,” 3 = “2-3 times a week,” 4 = “4-5 times a week,” and 5 = “Almost every day”) and changes in outdoor recreation level compared to pre-COVID level (1 = “Much less now than before COVID-19”, 2 = “Slightly less now than before COVID-19”, 3 = “About the same, 4 = “Slightly more now than before COVID-19”, and 5 = “Much more now than before COVID-19”).

#### COVID-specific risk and protective factors.

Social isolation was measured using a single item from the UCLA Loneliness Scale Version 3 [79]. Participants indicated how much the statement “I felt isolated” applied to them over the past two weeks using a 4-point scale (1 = “Not at all” to 4 = “Most of the time”).

Response categories for all subsequent items in this subsection were on a 6-point Likert scale (1 = “Disagree strongly”, 6 = “Agree strongly”). Perceived risk of infection was measured using a single item—participants indicated their agreement with the statement “There is a high likelihood of acquiring COVID-19 in general.” Beliefs about preventive measures were measured by having participants report their agreement with statements that concluded the sentence “COVID-19 preventative measures…,” including two items measuring positive beliefs (“…help lower the risk of infection”, “...help make our environment safer”) and three items measuring negative beliefs (“...are constraining,” “...cause anxiety in me,” and “...make it hard to live a full life”). The positive and negative belief scales showed acceptable internal reliability (Cronbach’s α =.73 and.82, respectively). Average scores were used for both. Precautionary behavior was measured by having participants report their agreement with the statement “I take active precautionary measures to lower the risk of infection.” Future outlook was measured by having participants report their agreement with separate statements measuring two items: vaccine outlook (“Situations are improving with the development of COVID-19 vaccines”) and COVID optimism (“I am optimistic that life will return to normal soon”).

#### Socio-demographics and COVID-related background.

Socio-demographic response options with ten or fewer responses were recoded into theoretically consistent groups or treated as missing. Participants reported the following socio-demographic characteristics: age (years), sex (male or female; with Trans/non-binary treated as missing due to low response rate), race (White, Asian, or Black or African American; with Native Hawaiian or Other Pacific Islander, American Indian or Alaska Native, and other treated as missing), work status (five categories recoded into three: work from home, work in public, and unemployed), parenting (yes or no), subjective financial condition (1 = “I’m behind on my bills”, 2 = “I’m barely making ends meet”, 3 = “I’m just getting by”, 4 = “I’m doing alright”, 5 = “I’m living comfortably”), and household income (measured from “19,999 or less” to “$150,000 or more” in increments of $10,000).

Participants also reported COVID-related background information, including their pre-existing medical conditions (yes or no) and COVID-19 infection status. “Confirmation via test,” and “Suspicion based on symptoms” were treated as “Infected”; “Neither” was treated as “Not infected”; “Prefer not to disclose” was treated as missing.

### Data analysis strategies

#### Sample characteristics and preliminary analysis.

Table 1 presents sample demographic characteristics. We confirmed sample representativeness using Chi-Square analysis by comparing 2015 American Community Survey data with proportions of key demographic attributes, including sex (χ^2^ = 0.004, df = 1, *p* =.947), age (in 10-year categories up to 60 years or older; χ^2^ = 5.346, df = 5, *p* =.375), and race (χ^2^ = 8.776, df = 5, *p* =.118). The percentage of participants who had been infected (12.1%) was also within 1% of cumulative confirmed infections at the time of the survey closing [52].

**Table 1:** Sample demographic and recreational characteristics (*n* = 503).

Variable		*n*	(%)	M	SD	Range
Age (Years)				46.6	16.14	20-79
Sex						
	Female	255	(50.6)			
	Male	241	(47.8)			
Race						
	White	370	(73.4)			
	Black or African American	70	(13.9)			
	Asian	40	(7.9)			
Work status						
	Work from home	214	(42.5)			
	Work outside home	92	(18.3)			
	Furloughed or unemployed	176	(34.9)			
Parenting						
	Yes	192	(38.1)			
	No	308	(61.1)			
Subjective financial condition						
	I’m behind on my bills	36	(7.1)			
	I’m barely making ends meet	62	(12.3)			
	I’m just getting by	153	(30.4)			
	I’m doing alright	172	(34.1)			
	I’m living comfortably	76	(15.1)			
Infection status						
	Confirmed or suspected	61	(12.1)			
	No	435	(86.3)			
Pre-existing medical condition						
	Yes	191	(37.9)			
	No	311	(61.7)			

Sample representativeness verification was conducted using Excel version 16.57. Power analyses for sample size were completed using G*Power version 3.1. All subsequent analyses were conducted using IBM SPSS 27. Complete cases were used for all analyses reflecting low missing rates (< 5% for all variables), which resulted in varied sample sizes. We also assessed linearity, normality, multicollinearity, and homoscedasticity for all regression analyses, with a correlation matrix for all variables used in the regression analysis provided in the supplement (S1 Table).

#### Outdoor activity coding and descriptive patterns.

Responses about “the most frequent outdoor activity” were coded via an iterative process informed by categorization frameworks used in existing studies [19,80]. Unique responses indicating the same activity using different wording were first merged (e.g., “biking” and “riding bike” were coded into “bicycling”). Activity types were then grouped into categories based on shared characteristics in resources, equipment, and level of physical exertion (e.g., hiking, walking). One researcher coded the open-ended responses into the activity categories. Subsequently, a second researcher used a list of those categories to independently code the same responses to determine interrater reliability, which was near-perfect (κ = 0.96, SE = 0.01, *p* <.001). Lastly, activity categories were grouped into one of two broad domains based on proximity to home and dependence on a natural resource or sport facility: (1) near-home outdoor activities and (2) outdoor sports and nature-based recreation.

We described outdoor recreation patterns for the full sample using proportions. Reflecting evidence supporting differences in outdoor recreation engagement by age, sex, race, and income [16,55], we analyzed differences in outdoor recreation engagement within these groups using nonparametric tests for categorical variables and Pearson correlation for continuous variables.

#### Regression analysis.

Ordinary least squares regression was used to analyze the associations between outdoor recreation engagement and perceived stress and well-being. Weighted least squares regression was used to estimate the depressive symptom model due to non-normality of residuals. For all regression analyses, changes in outdoor recreation engagement level was recoded into three levels (“Much less,” “About same,” and “Much more”) and treated as categorical, with “about same” serving as the reference group. Eight covariates (age, sex, race, work status, parental status, subjective financial condition, pre-existing medical conditions, and infection status) were included based on existing evidence supporting associations between these variables and mental health outcomes during COVID-19 [4,81,82]. All categorical variables (changes in outdoor recreation engagement level, sex, race, work status, parental status, pre-existing medical condition, and COVID-19 infection status) were dummy coded.

## Results

### Outdoor recreation engagement

Participants (*n* = 483) provided 183 unique open-ended responses about their “most frequent outdoor activity” during COVID-19, which were coded into 12 categories (Table 2). Most participants (56.9%) named walking as their most frequent activity, followed by hiking (8.3%), running (7.7%), and gardening (6.8%). Twice as many respondents reported a near-home activity (68.1%) than an outdoor sport or nature-based activity (31.9%).

**Table 2:** Most Frequent outdoor activities during COVID-19 (n = 483).

Variable			*n*	%
**Most Frequent Outdoor Recreation Activity** ^a^				
	*Near-Home Activities*		*329*	*68.1*
		Walking	275	56.9
		Gardening or yard work	33	6.8
		Sedentary or relaxing activities^b^	15	3.1
		Other^c^	6	1.2
	*Outdoor Sports and Nature-Based Recreation*		*154*	*31.9*
		Hiking^d^	40	8.3
		Running or jogging	37	7.7
		Bicycling	26	5.4
		Field or court sports	14	2.9
		Nature travel, visit, and photography^e^	14	2.9
		Water-based sports	13	2.7
		Ski, skate, and board sports	6	1.2
		Motorized recreation^f^	4	0.8
**Changes in Outdoor Recreation Level**				
	Much less now than before COVID-19		165	32.8
	Slightly less now than before COVID-19		122	24.3
	About the same		142	28.2
	Slightly more now than before COVID-19		50	9.9
	Much more now than before COVID-19		23	4.6
**Frequency of Outdoor Recreation**				
	Less than once a week		173	34.4
	Once a week		100	19.9
	2-3 times a week		123	24.5
	4-5 times a week		46	9.1
	Almost every day		60	11.9

^a^*n*=483, reflecting that *n* = 4 did not answer, *n* = 8 did not participate in outdoor recreation, and *n* = 8 responded with an activity that did not match the definition of outdoor recreation ^b^Included activities that are often sedentary (e.g., reading, hammocking, and tanning) or associated with relaxing (e.g., yoga, drinking, and picnicking) ^c^Included variations of playing with family or pets or on phone applications ^d^Included snowshoeing ^e^Included camping and visiting parks or natural areas ^f^Included ATV riding, motorcycling, and driving

Table 2 shows that over one-third (34%) of the sample recreated outdoors less than once a week, whereas 45% recreated outdoors one-to-three times a week and the remaining 21% recreated four-to-five times a week or more often. Most of the respondents (57%) reported reducing outdoor recreation to some degree during COVID-19, whereas 28% maintained their pre-COVID level and 15% increased engagement.

Table 3 presents demographic characteristics of outdoor recreation engagement. Several small but significant effects were observed: First, people who reported a near-home activity as their most frequent outdoor activity were on average almost seven years older than those who engaged in an outdoor sport or nature-based recreation as their most frequent outdoor activity (*p* <.001, η =.193). Age was positively correlated (*p* <.001) with outdoor recreation frequency. Second, outdoor recreation engagement relative to pre-COVID levels differed across racial groups (*p* =.002, Cramer’s V =.134). White participants were more likely to maintain their pre-COVID outdoor recreation level than Black or Asian participants, whereas the latter were more likely to reduce their outdoor engagement during COVID-19. White respondents also reported recreating outside more frequently than Black respondents (*p* =.025, η =.124). Third, people who perceived themselves as having a better financial condition recreated outside more frequently (r =.12, *p* =.009). The result also suggested significant differences in outdoor recreation frequency based on household income (*p* =.015, η =.144), but post-hoc tests did not reveal significant pairwise differences.

**Table 3:** Demographic characteristics of outdoor recreation engagement.

Variable	Most Frequent Outdoor Activity			Changes in Outdoor Recreation Level				Frequency of Outdoor Recreation	
	Near-home activity	Outdoor sport or nature-based recreation		Much less than pre-COVID	About same	Much more than pre-COVID			
**Continuous variables**	M (*SD*)	M (*SD*)	F-value (p-value) Eta (η)^a^	M (*SD*)	M (*SD*)	M (*SD*)	F-value (p-value) Eta (η)^a^	r (p-value)^b^	
Age	48.8 (16.5)	42.1 (14.7)	**18.60 (<.001),.193**	46.81 (15.7)	46.66 (16.5)	45.13 (14.5)	0.11 (.897)	**0.15 (<.001)**	
Subjective financial conditions	3.41 (1.1)	3.37 (1.1)	0.13 (.714)	3.29 (1.1)	3.41 (1.1)	3.7 (1.0)	1.58 (.206)	**0.12 (.009)**	
**Categorical variables**	*n* (row %)	*n* (row %)	X^2^ value (p-value) Cramer’s V ^a^	*n* (row %)^c^	*n* (row %)^c^	*n* (row %)^c^	X^2^ value (p-value) Cramer’s V ^a^	M (*SD*)^d^	F-value (p-value) Eta (η) ^a^
Sex									
Male	151 (64.5)	83 (35.5)	2.65 (.104)	71 (29.6)	160 (66.7)	9 (3.8)	3.14 (.208)	2.55 (1.33)	1.84 (.175)
Female	173 (71.5)	69 (28.5)		90 (35.3)	151 (59.2)	14 (5.5)		2.38 (1.38)	
Race									
White	250 (70.4)	105 (29.6)	4.23 (.120)	106 (28.7)^a^	248 (67.2)^a^	15 (4.1)^a^	**17.11 (.002),.134**	2.54 (1.39)^a^	**3.73 (.025),** **.124**
Black	38 (58.5)	27 (41.5)		33 (47.1)^b^	32 (45.7)^b^	5 (7.1)^a^		2.09 (1.15)^b^	
Asian	25 (62.5)	15 (37.5)		19 (47.5)^b^	18 (45.0)^b^	3 (7.5)^a^		2.28 (1.41)^a^^,^^b^	
Household Income									
<$29,999	83 (70.3)	35 (29.7)	2.87 (.412)	40 (33.1)	78 (64.5)	3 (2.5)	4.89 (.558)	2.12 (1.24)	**3.51 (.015),.144**
$30,000-69,999	107 (71.8)	42 (28.2)		47 (29.9)	103 (65.6)	7 (4.5)		2.58 (1.40)	
$70,000-99,999	84 (65.6)	44 (34.4)		45 (34.1)	81 (61.4)	6 (4.5)		2.43 (1.37)	
$100,000 or more	53 (62.4)	32 (37.6)		32 (36.0)	50 (56.2)	7 (7.9)		2.65 (1.36)	

^a^Effect sizes are provided if significant group differences existed (in bold) and interpreted per Cohen’s (1988) guideline.

^b^Significant correlations indicated in bold.

^c^Different superscripts indicate significantly different proportions using Multiple Comparison Bonferroni Correction.

^d^Different superscripts indicate significantly different means at *p* < 0.05 based on Scheffe post-hoc tests for equal variances

### Outdoor recreation and mental health during COVID-19

Table 4 provides regression analysis results for perceived stress, depressive symptoms, and well-being controlling for COVID-specific protective and risk factors, demographics, and COVID-related background (*n* = 458).

**Table 4:** Linear regression models for perceived stress, depressive symptoms, and well-being during COVID-19 (*n* = 458).

	Perceived Stress				Depressive Symptoms				Well-being			
	*B*	*SE*	*β*	∆*R*_j_	*B*	*SE*	*β*	∆*R*_j_	*B*	*SE*	*β*	∆*R*_j_
*Outdoor recreation engagement*				.01				0.04				0.08
Frequency	0.12	0.15	0.03		0.01	0.04	0.01		0.77^***^	0.20	0.18^***^	
Much less than pre-COVID (about same = ref.)	0.85^*^	0.42	0.08^*^		0.35^**^	0.13	0.10^**^		-1.05	0.58	-0.08	
Much more than pre-COVID (about same = ref.)	-0.74	0.83	-0.03		-0.10	0.17	-0.02		-0.59	1.13	-0.02	
*COVID-specific factors*				.35				.38				.22
Social isolation	1.98^***^	0.19	0.41^***^		0.77^***^	0.07	0.46^***^		-1.88^***^	0.26	-0.32^***^	
Perceived risk of infection	0.34^*^	0.15	0.09^*^		0.00	0.03	0.00		-0.15	0.20	-0.03	
Negative beliefs about preventive measures	0.56^***^	0.16	0.14^***^		0.13^**^	0.04	0.12^**^		-0.38	0.21	-0.08	
Positive beliefs about preventive measures	0.15	0.20	0.03		0.05	0.05	0.05		-0.11	0.27	-0.02	
Precautionary behavior	-0.48^*^	0.24	-0.09^*^		-0.02	0.05	-0.02		0.21	0.32	0.03	
Vaccine outlook	0.22	0.18	0.05		0.00	0.04	0.00		-0.25	0.25	-0.05	
COVID optimism	-0.15	0.14	-0.04		-0.07^*^	0.04	-0.08^*^		0.82^***^	0.19	0.20^***^	
*Socio-demographics and COVID Background*				.10				.06				.04
Age	-0.10^***^	0.01	-0.33^***^		-0.016^*^	0.00	-0.21^***^		0.04^*^	0.02	0.10^*^	
Male (female = ref.)	-0.42	0.36	-0.04		0.04	0.09	0.02		0.92	0.49	0.08	
Asian (White = ref.)	-1.74^**^	0.67	-0.09^**^		-0.54^***^	0.13	-0.16^***^		0.65^**^	0.23	0.12^**^	
Black (White = ref.)	-0.66	0.52	-0.05		-0.23	0.14	-0.06		-0.12	0.49	-0.01	
Subjective financial condition	-0.33^*^	0.17	-0.07^*^		-0.01^*^	0.04	-0.084^*^		1.95^*^	0.91	0.09^*^	
Work in Public (work from home = ref.)	-0.23	0.48	-0.02		0.14	0.12	0.05		0.36	0.71	0.02	
Unemployed (work from home = ref.)	-0.43	0.39	-0.04		0.23^*^	0.11	0.085^*^		0.11	0.65	0.01	
Parenting (not parenting = ref.)	1.06^**^	0.36	0.11^**^		0.01	0.09	0.01		-0.39	0.53	-0.03	
Infected (not infected = ref.)	1.14^*^	0.54	0.07^*^		0.36	0.20	0.06		-0.94	0.73	-0.05	
Pre-existing medical condition (no = ref.)	0.49	0.39	0.05		0.38^***^	0.11	0.13^***^		-1.27^*^	0.54	-0.10^*^	
Cumulative Adjusted R^2^	.46				.48				.33			
F-value	20.60				22.25				24.07			
F-test *p*-value	<.001				<.001				<.001			

*B*: unstandardized coefficient; *SE*: standard error, *β*: standardized coefficient; *∆R*_*j*_: change in adjusted R-square;

* *p*-value < 0.05;

** *p*-value < 0.01;

*** *p*-value < 0.001.

#### Outdoor recreation and perceived stress.

Decreased outdoor recreation engagement predicted higher perceived stress (.85 points higher on a 21-point scale than the maintenance group, *p* =.046). Neither frequency nor increasing outdoor recreation engagement were significant predictors. Outdoor recreation variables collectively explained 1.3% of the variance in perceived stress.

Three COVID-specific risk factors predicted significantly higher perceived stress, including social isolation (*β* = 0.41, *p* <.001), negative beliefs about preventive measures (*β* = 0.14, *p* <.001), and perceived risk of infection (*β* = 0.09, *p* =.02). Older age (*β* = -0.33, *p* <.001) reporting Asian race (relative to White race, *β* = -0.09, *p* =.01) and better subjective financial condition (*β* = -0.07, *p* =.049) predicted lower perceived stress, while parenting (*β* = 0.11, *p* =.004) and having a COVID-19 infection (*β* = 0.07, *p* <.036) predicted higher perceived stress. The full model accounted for 46.1% of the total variance in perceived stress.

#### Outdoor recreation and depressive symptoms.

Outdoor recreation’s effects on depressive symptoms were similar to the effects observed in the perceived stress model. Decreased outdoor engagement was the only significant predictor of depressive symptoms (.35 points higher on a 7-point scale than the maintenance group, *p* =.007). Frequency and increased outdoor recreation engagement were not significant. Outdoor recreation engagement variables accounted for 4.2% of the variance in depressive symptoms.

Stronger social isolation (*β* = 0.46, *p* <.001) and negative beliefs about preventive measures (*β* = 0.12, *p* =.002) predicted higher depressive symptoms, while a more optimistic outlook about the pandemic (*β* = -0.08, *p* =.046) predicted lower depressive symptoms. Older age (*β* = -0.21, *p* <.001), better subjective financial condition (*β* = -0.08, *p* =.024), and reporting Asian race (relative to White, *β* = -0.16, *p* <.001) predicted lower depressive symptoms, while having a pre-existing medical condition (*β* = 0.13, *p* =.001) or being unemployed (relative to working from home, *β* = 0.08, *p* =.034) predicted higher depressive symptoms. The full model accounted for 48.3% of the total variance in depressive symptoms.

#### Outdoor recreation and well-being.

More frequent outdoor recreation was associated with higher well-being (*β* = 0.18, *p* <.001). Changes in engagement levels were not significant predictors. Outdoor recreation engagement variables accounted for 7.7% of the variance in well-being.

Higher social isolation (*β* = -0.32, *p* <.001) predicted lower well-being, while COVID optimism (*β* = 0.20, *p* <.001) predicted higher well-being. Better subjective financial condition (*β* = 0.12, *p* =.004), older age (*β* = 0.10, *p* =.027), and reporting Asian race (relative to White*, β* = 0.09, *p* =.033) were associated with higher well-being, while having a pre-existing medical condition (*β* = -.10, *p* =.018) predicted lower well-being. The whole model accounted for 33.1% of the total variance in well-being.

## Discussion

In this study, we described patterns of outdoor recreation among U.S. adults during the height of the COVID-19 pandemic and examined how relative and cumulative engagement in outdoor recreation related to mental health and well-being. We observed widespread reductions in outdoor recreation levels relative to the pre-COVID period. Our data also suggested distinct relations between different measures of outdoor engagement and mental health, wherein relative measures were better predictors of negative mental health, while cumulative frequency measures better predicted well-being. Detailed discussions of results and implications follow.

### Outdoor recreation engagement during COVID-19

More than 45% of our sample reported recreating outside at least once a week. While this rate is higher than prior findings [55], it is likely due to the inclusion of a broader set of outdoor activities in this study, including non-nature-based near-home activities and those involving low levels of physical exertion (e.g., walking, sedentary and relaxing activities). Our finding of more frequent near-home activities relative to traditional outdoor sports and nature-based recreation extended similar observations from the lockdown period to a later phase [43], accentuating the role of nearby natural spaces and non-tradition outdoor activities as people continued to adapt to pandemic challenges [47,83,84]. This finding revealed that in the face of disruptions people fell back on low-barrier outdoor activities [83] as they navigated and negotiated various constraints such as travel restrictions, park closures, crowding, and risk of infections [43,85].

Our results also suggested widespread (57%) reductions in outdoor recreation (relative to pre-COVID level) despite a general increase of leisure time [86], relative availability of outdoor space (compared to indoor spaces), and public health guidance that encouraged using outdoor space for physical activity and socialization [87]. Several reasons might have contributed to the observed decreases in outdoor engagement: negative impacts of increasing COVID-19 cases [34], people returning to other activities during later phases of the pandemic [39], and disengagement of racial minorities, possibly due to a lower sense of belonging or access [16,88]. Our data provided empirical evidence aligned with these explanations. Despite mixed evidence in outdoor recreation participation rates during later phases or after the pandemic, recreation managers and public health policymakers should aim to minimize restrictions on and barriers to these spaces to prevent decreased engagement. They should also consider incentives to facilitate engagement, such as lowering costs and improving transportation to facilities, particularly for low-income groups [35,39].

We observed significant differences in outdoor recreation engagement based on age, race, and subjective financial condition. With respect to cumulative exposure, we observed more frequent outdoor activities among older adults, possibly reflecting the higher levels of free time available to retired adults. However, we observed no age differences in relative changes in outdoor recreation engagement compared to pre-pandemic levels, contrasting findings in Vienna where younger adults were more likely to increase outdoor recreation participation [51]. Consistent with previous studies, we observed a stronger preference in near-home recreation activities among older adults [11,89].

Additionally, we observed less frequent and reduced outdoor recreation among people of color and those who perceived themselves as being in a poorer financial condition, possibly due to historically greater constraints to nature access [90, 91] and heightened barriers to outdoor space use during the pandemic among these groups [92, 93]. In light of these findings, we suggest potential increased investment in park-poor areas by municipalities [93]. There are practical measures for implementing standards for park and green space access, such as the 3-30-300 rule, which encourages city designers to ensure that every dwelling has three visible trees from its windows, every neighborhood has 30% tree canopy, and every home is 300 meters to a park or publicly accessible greenspace [94]. Adopting these measures or similar alternatives will facilitate wholistic accounts of health benefits derived from green views and park space, maximizing outdoor recreation and related facilities’ potential as a means to equitably promote healthy behavior and distribute health infrastructure [94].

Lower levels of outdoor recreation engagement, especially among younger adults and those who reported poorer financial well-being, is concerning as mounting evidence [4,82] suggested heightened risk for poor mental health and well-being for these groups during the pandemic. Future empirical studies should examine potential constraints at the intrapersonal, interpersonal, and structural levels that may have contributed to the overall decreased engagement in outdoor activities [95, 96]. There is also an opportunity to examine causes of inequities in outdoor engagement between people of different races, ethnicities, and income backgrounds during COVID-19 and their enduring impacts on recreation participation as they relate to identity, perception, and experience [88,97] or place-based factors [15,98].

### Outdoor recreation and mental health

Our result suggested that different measures of outdoor engagement predicted different aspects of mental health. Specifically, while frequency of outdoor recreation predicted well-being, only reduced engagement relative to pre-COVID level was significantly associated with perceived stress and depressive symptoms. Past studies reported similar effects of relative changes in outdoor engagement without controlling for frequency [23,24,48,56]. By including both types of measures, our study illustrated the unique effect of failing to maintain or increase outdoor engagement on mental health. Echoing prior research [62], we suggest that adaptive engagement plays a more prominent role than cumulative exposure in mitigating the adverse effect of negative events on mental health. Combined, these findings provide a nuanced insight into the role of outdoor activity, and nature contact in general, in aiding stress recovery in contexts marked by significant changes and constraints [99–101]. Future research should be more mindful of what types of measures to use when studying context-specific health outcomes of leisure or outdoor recreation.

Prior research has suggested that spending more time outdoors may increase people’s well-being by increasing their connection to nature, which in turn promotes continued engagement and restoration [8,19,22,49]. Our findings supported outdoor recreation’s positive effect on well-being controlling for COVID-specific contextual factors, with frequency emerging as one of the strongest protective factors. Collectively, outdoor recreation variables predicted well-being better than they predicted negative mental health, providing additional support for leisure’s differential associations with positive and negative mental health [62].

Our regression models explained a significant portion (33–48%) of the variance in mental health outcomes, with all statistically significant risk and protective factors exhibiting anticipated effects. As such, they provide a robust framework for future research in similar contexts characterized by marked stressors and disruptions. Notably, among all included variables, social isolation emerged as the strongest predictor of mental health across all three models.

Researchers have suggested that nature-based activities, when engaged in as shared activities, provide opportunities for social connections and relationship building, which in turn accrue social support and reduce loneliness [102,103]. This could be especially true in the context of COVID-19, wherein outdoor space was perceived as relatively safe and instrumental for social interactions [51,104]. However, facilitating outdoor engagement and interactions also requires balancing heightened risk for infections in future similar public health crises [9,87] and other potential negative impacts of crowding on both the recreators and natural resources. Future research may examine the potential mediating effect of social isolation as a mechanism for explaining outdoor recreation’s positive impact on well-being during the pandemic and beyond.

Our results contributed to the growing evidence for outdoor recreation’s role as an important health behavior [20,105]. Parks and other outdoor recreation spaces play a critical role in supporting this health behavior, with the potential to reduce inequalities in wide-ranging negative health outcomes [17]. Corresponding to the increasing perceived importance of parks and outdoor spaces to public health post-pandemic [106,107], we believe it is crucial to maintain or even increase access to outdoor recreation spaces during future crises like the COVID-19 pandemic, especially in park-poor areas or communities where vulnerable groups concentrate. In addition to promoting outdoor recreation as a restorative, relatively low-effort health behavior, governments could systematically invest in green infrastructure as a preventative health measure while addressing broader aims like promoting sustainability [94,107]. However, evidence suggests that individual adaptative strategies during COVID-19, including increasing effort and adjusting the time of recreation, were strong predictors of outdoor recreation above and beyond structural factors like park access [108]. Therefore, land managers, practitioners, and advocates could invest in efforts that facilitate or encourage adaptative outdoor recreation behaviors during future stressful events. The opportunity to help maintain engagement in low-barrier and less specialized outdoor recreation activities (e.g., walking) among new and existing participants also remains important [34,47], as is facilitating reintroduction to outdoor recreation among people who stopped during the pandemic [15,88].

### Strengths and limitations

We collected data through an online survey. While the use of a crowd-sourcing platform enabled quick recruitment and participant screening, it also introduced potential self-selection bias at the platform and survey level as is often the case for other types of digital user-generated data [109]. These biases were partially mitigated by recruiting a sample representative of the general adult population in key demographic variables. Nonetheless, our study sample was not a random sample, and caution should be heeded when generalizing the findings. Furthermore, while our sample size was consistent with related literature [30] and suitable for measuring population-level effects based on power analysis, a larger sample size may have been able to better detect within-group differences and smaller effects.

Due to the cross-sectional nature of our data, causality can only be tentatively inferred based on theory and prior evidence. A longitudinal design would be instrumental in addressing this limitation by tracking the outdoor recreation-mental health relationship through a stressful event while accounting for potential confounders [48]. Specifically, our results may be susceptible to two issues: (1) seasonal effects, as the generally low outdoor recreation participation rates during winter could mask our findings [37], and (2) self-report bias associated with retrospective reports, which may be overcome by measuring real-time engagement.

## Conclusion

The mental health benefits of nature exposure are well established [25,92], particularly as a result of the interplay between psycho-physiological effects from viewing natural features, increased physical activity, and fostered social interactions [18,99,100]. As a primary and direct form of nature contact, outdoor recreation provides an important pathway for maintaining or improving human health and well-being. In the context of COVID-19, outdoor recreation was most frequently performed in the convenience and relative safety of near-home outdoor spaces. This study highlights the critical role of adaptive outdoor engagement in protecting against poor mental health and the importance of frequent outdoor recreation in maintaining well-being. Meanwhile, our observation of a common reduction in outdoor recreation among U.S. adults during the pandemic, particularly among racial minorities, younger adults, and people perceiving worse financial conditions, raises concerns about the persisting effect of structural inequity in people’s ability to engage in outdoor recreation as a health behavior. We urge researchers, policymakers, and land managers to invest in promoting outdoor recreation engagement as vectors for health equity, including facilitating the maintenance of outdoor recreation during future enduring crises or similar events.

## Supporting information

S1 Table Correlation matrix of all variables included in linear regression models examining mental health. Outdoor recreation was abbreviated to OR. Dichotomous variables were dummy coded. *p-value < 0.05; **p-value < 0.01; ***p-value < 0.001.
(DOCX)
