# Supplementary material for: Outdoor recreation’s association with mental health and well-being during the COVID-19 pandemic
Source: PLoS One. 2025 Apr 17;20(4):e0321278. doi: 10.1371/journal.pone.0321278 (PMC12005518; doi:10.1371/journal.pone.0321278)
Supplement: S1 Table — Correlation matrix of all variables included in linear regression models examining mental health. Outdoor recreation was abbreviated to OR. Dichotomous variables were dummy coded. *p-value < 0.05; **p-value < 0.01; ***p-value < 0.001. (DOCX) [file pone.0321278.s001.docx]

S1 Table: Correlation matrix of all variables included in linear regression models examining mental health.

| Perceived Stress | - |  |  |  |  |  |  |  |  |  |  |  |  |  |  |  |  |  |  |  |  |  |
| --- | --- | --- | --- | --- | --- | --- | --- | --- | --- | --- | --- | --- | --- | --- | --- | --- | --- | --- | --- | --- | --- | --- |
| Depressive symptoms | .67^**^ | - |  |  |  |  |  |  |  |  |  |  |  |  |  |  |  |  |  |  |  |  |
| Well-being | -.53^**^ | -.64^**^ | - |  |  |  |  |  |  |  |  |  |  |  |  |  |  |  |  |  |  |  |
| OR frequency | -0.12^**^ | -.16^**^ | .28^**^ | - |  |  |  |  |  |  |  |  |  |  |  |  |  |  |  |  |  |  |
| Much less OR | .13^**^ | .15^**^ | -.22^**^ | -.41^**^ | - |  |  |  |  |  |  |  |  |  |  |  |  |  |  |  |  |  |
| Much more OR | -.06 | -.05 | .06 | .22^**^ | -.15^**^ | - |  |  |  |  |  |  |  |  |  |  |  |  |  |  |  |  |
| Social isolation | .59^**^ | .61^**^ | -.46^**^ | -.11^*^ | .09^*^ | -.02 | - |  |  |  |  |  |  |  |  |  |  |  |  |  |  |  |
| Perceived risk of infection | .17^**^ | .09^*^ | -.12^**^ | -.04 | .06 | .00 | .13^**^ | - |  |  |  |  |  |  |  |  |  |  |  |  |  |  |
| Neg. preventive measures | .23^**^ | .21^**^ | -.16^**^ | -.06 | 0.10^*^ | -.06 | 0.22^**^ | -.09^*^ | - |  |  |  |  |  |  |  |  |  |  |  |  |  |
| Pos. preventive measures | .03 | .04 | -.02 | .02 | .01 | .07 | .07 | .35^**^ | -.31^**^ | - |  |  |  |  |  |  |  |  |  |  |  |  |
| Precautionary behavior | -.10^*^ | -.06 | .02 | .03 | .12^**^ | .00 | .01 | .30^**^ | -.21^**^ | .51^**^ | - |  |  |  |  |  |  |  |  |  |  |  |
| COVID outlook | .02 | -.01 | .06 | .09^*^ | -.01 | .03 | .07 | .18^**^ | -.05 | .31^**^ | .23^**^ | - |  |  |  |  |  |  |  |  |  |  |
| Vaccine optimism | -.05 | -.09 | .21^**^ | .04 | .00 | .03 | -.02 | -.02 | -.04 | .15^**^ | .01 | .45^**^ | - |  |  |  |  |  |  |  |  |  |
| Age | -.43^**^ | -.31^**^ | .22^**^ | .15^**^ | .01 | -.02 | -.29^**^ | -.11^*^ | .02 | -.05 | .20^**^ | .10^*^ | .01 | - |  |  |  |  |  |  |  |  |
| Sex | -.05 | -.05 | .13^**^ | .06 | -.06 | -.04 | .00 | -.16^**^ | .02 | -.10^*^ | -.18^**^ | .12^**^ | .13^**^ | -.03 | - |  |  |  |  |  |  |  |
| Asian | -.02 | -.03 | .06 | -.04 | .09^*^ | .04 | .02 | .07 | -.02 | .04 | .00 | -.02 | -.02 | -.11^*^ | -.01 | - |  |  |  |  |  |  |
| Black | -.05 | -.07 | .02 | .11^*^ | .12^**^ | .05 | -.09^*^ | -.02 | -.09 | .04 | -.05 | -.08 | .09 | -.10^*^ | -.01 | -.12^**^ | - |  |  |  |  |  |
| Sub. financial well-being | -.25^**^ | -.27^**^ | .28^**^ | .12^**^ | -.06 | .06 | -.24^**^ | -.05 | -.01 | .02 | .07 | .14^**^ | .07 | .17^**^ | .06 | .09 | -.08 | - |  |  |  |  |
| Working outside home | .02 | .01 | .02 | -.03 | .00 | .02 | -.04 | .00 | .01 | -.04 | -.14^**^ | -.04 | -.03 | -.16^**^ | .08 | .02 | .04 | -.03 | - |  |  |  |
| Unemployed | -.03 | .08 | -.10^*^ | -.08 | -.03 | -.09 | .08 | .03 | -.03 | .01 | .02 | -.03 | -.04 | .14^**^ | -.05 | -.08 | -.06 | 0.10^*^ | -.37^**^ | - |  |  |
| Parenting | -.05 | -.11^*^ | .09^*^ | .04 | .08 | -.06 | -.15^**^ | -.04 | .04 | -.11^*^ | -.01 | .01 | .08 | .25^**^ | -.05 | .00 | .03 | .09^*^ | -.04 | .03 | - |  |
| Infection status | .16^**^ | .14^**^ | -.12^*^ | -.06 | .08 | -.05 | .10^*^ | .12^**^ | -.02 | .02 | -.02 | .03 | -.01 | -.07 | .04 | -.06 | .07 | -.04 | .02 | .01 | .00 | - |
| Pre-existing condition | .10^*^ | .17^**^ | -.21^**^ | -.04 | .12^**^ | -.13^**^ | .16^**^ | .13^**^ | -.01 | .10^*^ | .16^**^ | .02 | -.21^**^ | .21^**^ | -.09^*^ | -.06 | -.13^**^ | -.15^**^ | -.12^**^ | .10^*^ | .00 | .10^*^ |
|  | Perceived stress | Depressive symptoms | Mental well-being | OR frequency | Much less OR | Much more OR | Social isolation | Perceived risk of infection | Neg. preventive measures | Pos. preventive measures | Precautionary behavior | COVID outlook | Vaccine optimism | Age | Sex | Asian | Black | Sub. financial well-being | Working outside home | Unemployed | Parenting | Infection status |

Notes: Outdoor recreation was abbreviated to OR. Dichotomous variables were dummy coded. ^*^*p*-value < 0.05; ^**^*p*-value < 0.01; ^***^*p*-value < 0.001.
